# Narrowing the gap between eye care needs and service provision: a model to dynamically regulate the flow of personnel through a multiple entry and exit training programme

**DOI:** 10.1186/1478-4491-7-42

**Published:** 2009-05-29

**Authors:** Keith Masnick

**Affiliations:** 1 Institute for Eye Research and School of Public Health and Community Medicine, University of New South Wales, Kensington, Australia

## Abstract

**Background:**

The purpose of this paper is to present a complex yet transparent, computable model to simulate the regulation of the flow of personnel through a previously described multiple-entry, multiple-exit eye care training scheme linked to the health workforce. This methodology should be a useful tool for the planner; it can address changes and feedbacks over time and be sensitive to any unexpected consequences of the interactions. The same model template can be applied to calculate the finances associated with the personnel flow.

**Presentation of the hypothesis:**

The worth of any model or set of concepts of human resources for health is considerably enhanced by actual field application. However, implementation involves the selection of one set of parameters and a large, long-term commitment of resources. A far less expensive and time-consuming, yet still effective, method of testing assumptions and ideas would be to simulate their application using a variety of possible inputs, structural configurations and/or desired outcomes. To that end, this paper presents a computable, dynamic model of personnel flows within a health system.

**Testing the hypothesis:**

Some testing of the model has been demonstrated in a previous paper. However, the value of the model is that all stakeholders can enter their own data and parameter assumptions and readily review the outcomes.

**Implications of the hypothesis:**

The complex yet easy-to-use model presented in this paper opens the debate on current and future policy to any stakeholder. A very wide range of scenarios can be considered and a selected option can be monitored and changed dynamically over time.

## Background

Implementation of a model of human resource dynamics within a health system is a time- and resource-consuming project. Changes are often difficult to accommodate, and bring with them both intended and unintended results. In addition, there is always the question of whether there might be better solutions to the situation in both the short and long term.

To address this issue, considerable effectiveness can come from mathematically simulating the model. This will allow for examination of a number of scenarios and the ability to include variables that may be unimportant initially but be influential later in the programme.

This paper provides an example of the application of a relevant simulation to a previously proposed needs-based eye care training system [1] that is directly related to positions in a developing country's health scheme. This training programme would be more efficient, responsive and cost-effective than the normal training plan by occupation.

At the outset it should be recognized that modelling is but one tool to be used in the final decision of the structure of an appropriate eye care system. It is beyond this paper's scope to include discussions of complex interprofessional and political interplay.

The proposed training system is based on the notion that all practitioners within eye care be trained with a selection of topics from a common set of competences. The variables in the training system were the number of practitioners at specific occupational levels, their time to train and the standard at which each competence would be applied to each student. In theory this would allow any practitioner to increase his or her range of competences by both acquiring new ones and by lifting the standard of those already attained. The advantage of this mobility is that it injects a faster, less expensive and more flexible component into workforce planning and avoids duplications.

## Presentation of the hypothesis

In order to meet the varying needs of the eye care system, a multiple entry and exit training programme (MEES) was proposed whereby students leave training with a specific set of competences at a number of predefined exits. They can either remain at that level in the workforce or return at a later stage to acquire a higher group of competences to meet health system demands.

**Figure 1:**
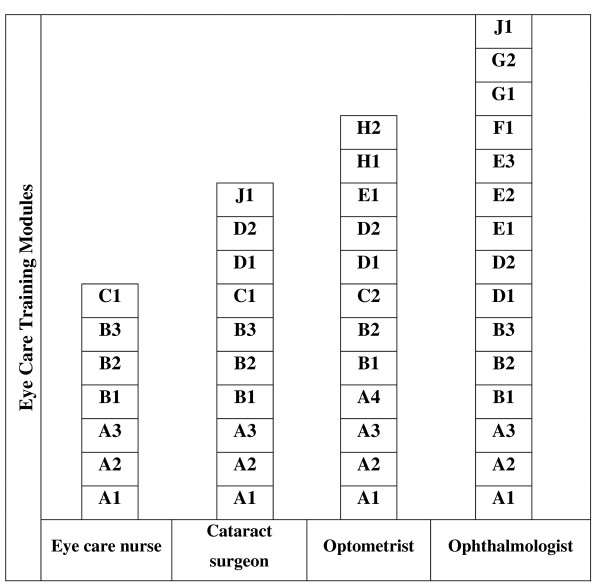
**Example of the use of a proposed modular training system for production of eye care personnel**.

Common access to competences permits movement across the normal professional training silos which increases the flexibility of the training system to respond to changes in the health service and improves understanding and respect across the range of health workers. For example, as shown in Fig. 1, a student who has completed the training elements for an Eye Care Nurse could become an optometrist by adding the training modules A4 and those from C2 to H2.

Within reason, these additional modules can be learnt some time after the completion of the original set of modules. In the case of eye care, this permits the definition of a new role of non-physician cataract surgeons based on the competences of optometrists, nurses or similarly trained clinical officers.

An essential step in improving the flow of students through a MEES is estimating the initial number of students and the proportion returning at each stage of higher learning, as well as the number of graduates entering the workforce at each stage.

Traditionally, these estimates are formed from the collective opinions of experts. However, the system is inherently complex and constantly changing over time. It becomes quite difficult, if not impossible, for a planner to integrate all the interactions in a meaningful way.

To overcome this problem and to reduce uncertainty, a computable dynamic model is proposed from which a number of scenarios can be played out over time in order to show the effects of a range of assumptions and estimates. This type of modelling is a synthesis of a large number of small units of activity and how each relates to and interacts with its immediate neighbours and the system as a whole. The inclusion of feedback and temporal features makes the model dynamic and non-linear rather than static and linear.

## Testing the hypothesis

### Building the simulation components of the MEES model

**Figure 2:**
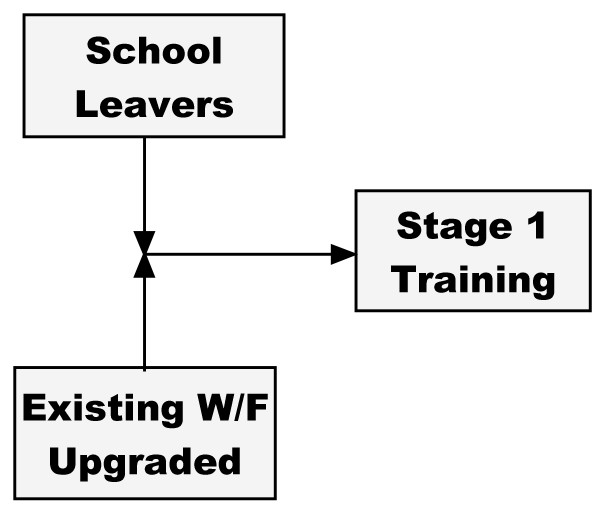
**Entrants into Stage 1 of training**.

The proposed plan uses a stock (a group of identical individuals) and flow (the processes that change the level of a stock) model. As a simple illustrative example, this simulation uses only the four stages of optometry training. Nurse, ophthalmology and community working modules complete the model but are not part of the immediate simulation. Figure 2 shows a stock of school leavers entering Stage 1 of training, together with existing workers whose skills have been sufficiently upgraded.

**Figure 3:**
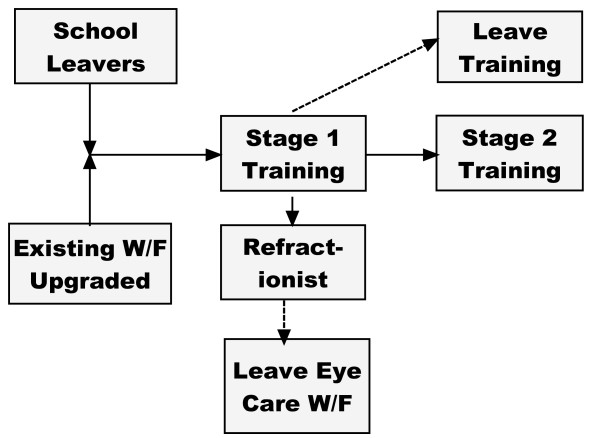
**Options for graduates of Stage1 training**.

At the end of the first year, graduates can either enter the workforce as refractionists, move onto Stage 2 of training or will have dropped out of the course. Some existing refractionists will retire (Fig. 3).

**Figure 4:**
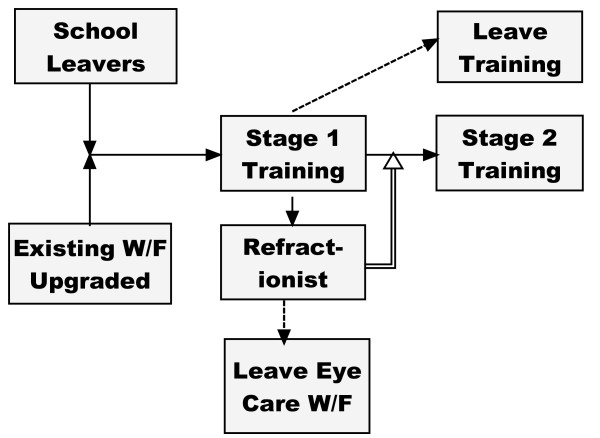
**Entrants into Stage 2 training**.

Entry into Stage 2 will come from graduates who have immediately completed Stage 1 and from the stock of existing refractionists (Fig. 4).

**Figure 5:**
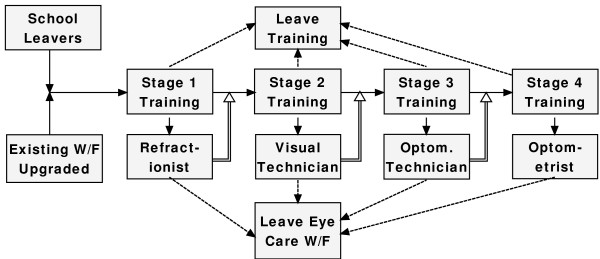
**Training and workforce flow for the optometry sector of eye care personnel**.

This process shown in Fig. 4 is repeated for the four stages of training (Fig. 5).

**Figure 6:**
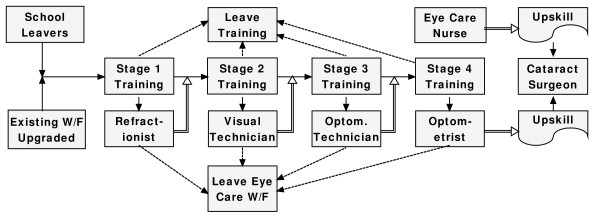
**Training and workforce flow for the non-physician sector of eye care personnel**.

To complete the non-physician sector of eye care personnel deployment and training, eye care nurses have been added. Both eye care nurses and optometrists can add further training modules that will allow them to train as cataract surgeons (Fig. 6). To keep the model from becoming over-complex, it has been assumed that the number of graduates at any level is below the optimum number required for the workforce. Similarly, *Eye Care Nurse *is shown as a stock (trained personnel – retirements).

To make the flows in Fig. 6 computable, iThink system dynamics modelling software (ISEE Systems) has been used. iThink has the advantage over spreadsheets in that each individual flow can be readily visualized in the context of the overall system structure and tested for both its local and global effects. While spreadsheets show the results of calculations, drilling down to the assumptions and supporting stock/flow structure, equations and data is more difficult than with iThink.

**Figure 7:**
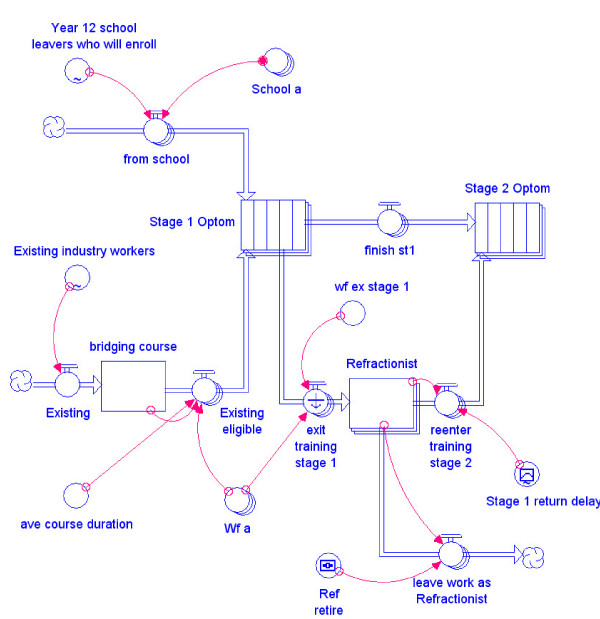
**Entry into an eye care multiple entry and exit training scheme**.

Figure 7 illustrates the iThink rendering of Fig. 4. The actual number of personnel involved in each of these flows is calculated by using conditions within the converters (circles) attached to flow taps by connectors (curved arrows). For example, the converters labelled *Year 12 school leavers who will enrol *and *Existing industry workers *determine how many school leavers flow into stage 1 of the optometry programme. Converters can either be constant (the same number in each period) or can be determined by a formula that reflects changing conditions.

**Figure 8:**
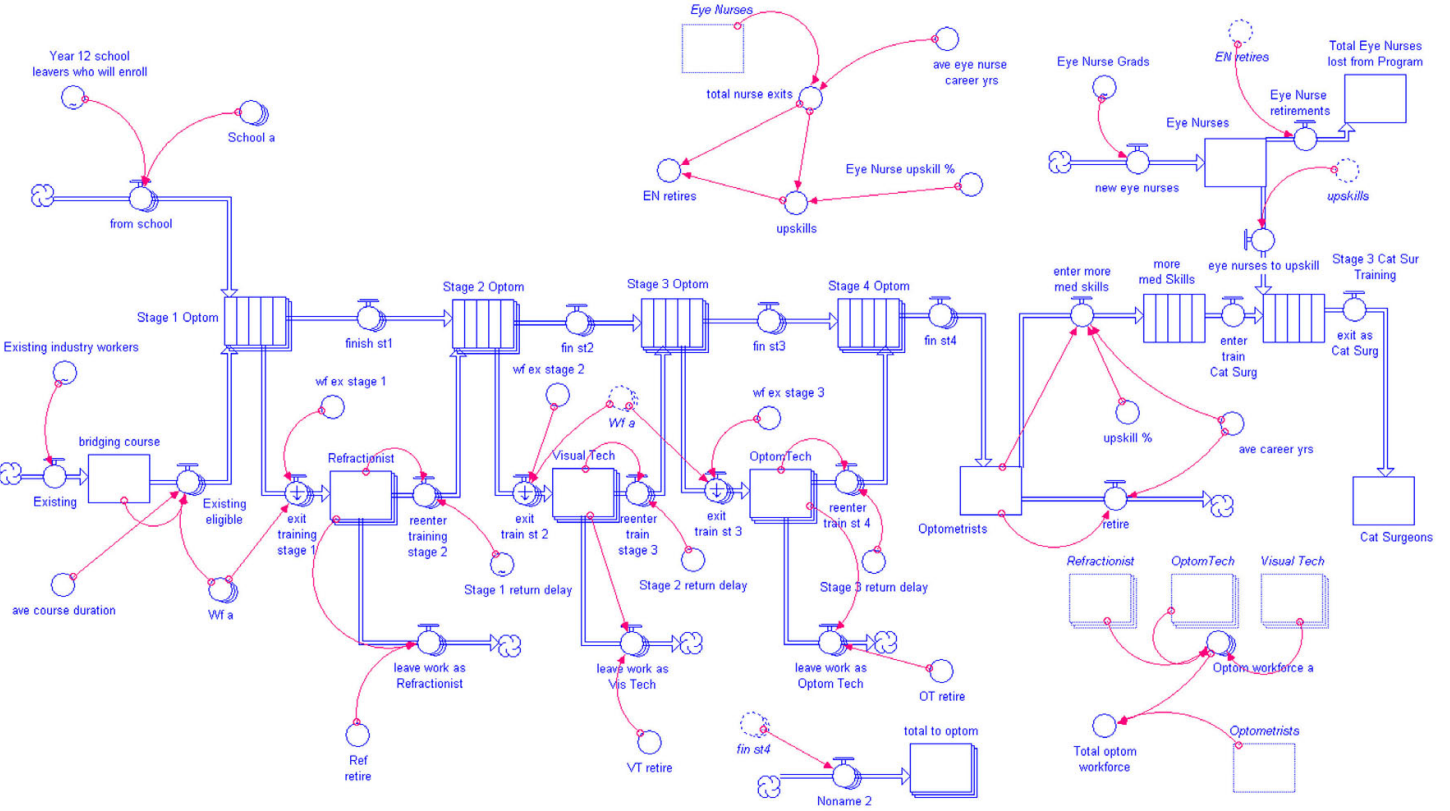
**The computable model of an eye care MEES showing the stocks and flows of personnel through training and into and out of the workforce**.

Figure 8 shows the complete iThink model of the non-physician sector of eye care. Using a computable model makes it possible to simulate a range of scenarios to predict the dynamic effects of the assumptions introduced into the model.

### Two scenarios

**Figure 9:**
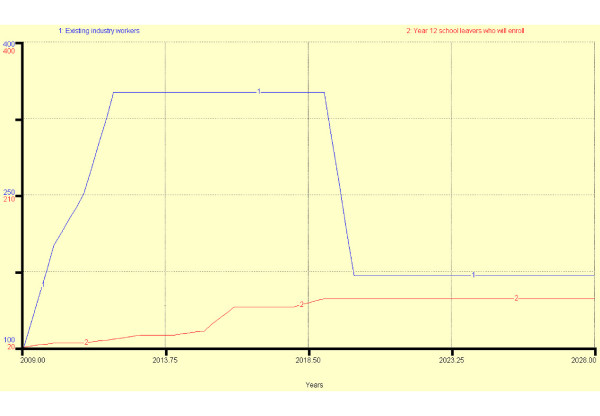
**Personnel enter and leaving training and the workforce 2009 – 2028**.

**Figure 10:**
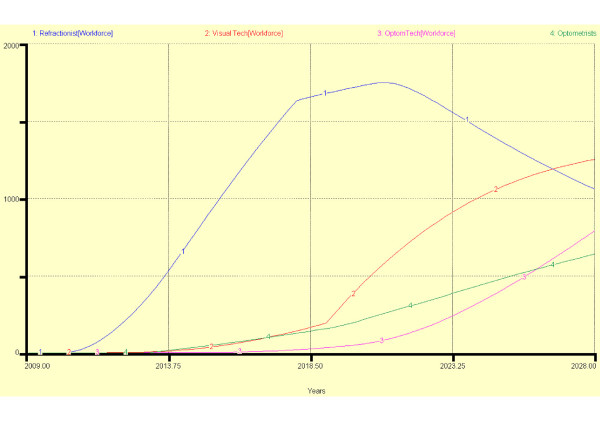
**Scenario 1 of personnel flow through a MEES model**.

**Figure 11:**
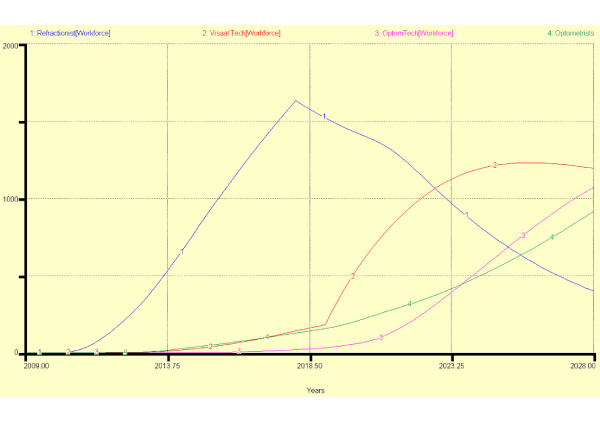
**Scenario 2 showing an accelerated personnel flow through a MEES model**.

Figure 9 shows the source of entrants into the programme between 2009 and 2028. Figures 10 and 11 show two different scenarios in which it has been assumed that all school leavers move through the stages without failure or leaving; that all *existing industry workers *enter the workforce at the end of each stage of training and entrants after 2018 must progress to stage 2. In scenario 1 after two periods (years) in the workforce at each exit point, 10% of *refractionists *re-enter stage 2 training, 25% of *visual technicians *re-enter stage 3 training but no *optometry technicians *enter final optometrist training. In scenario 2, in order to encourage a higher standard of service, after 2018, 30% of refractionists re-enter stage 2 training, 40% of visual technicians re-enter stage 3 training and 25% of *optometry technicians *train to become *optometrists*. The result of the new policy change is that there are fewer *refractionists *but more *optometry technicians *and *optometrists*.

### Other complementary models

The example shown has a number of complementary models. The existing workforce shown in Fig. 2 can be readily expanded to included community health and basic eye care workers. Similarly, graduates at any the exit points shown in Fig. 6 can enhance their skills in many subspecialties.

At a later date, the financial data associated with the personnel flows will be addressed by adding the costs associated with those flows or states and estimating the benefits or effects of the personnel numbers. This is essentially an activity-based costing method.

## Implications of the hypothesis

Simulation of a model is an effective and efficient tool that should be used with all human resource models. To show its application, a computable model has been presented to describe the flow of personnel though a multiple-entry, multiple-exit training scheme and thence into the health workforce. The model presented allows a planner to integrate accessible yet complex interactions by simulating and compensating for the effects over time of a range of differing scenarios. By understanding complexity and the environment within which the model will operate, intended and unintended consequences can be observed and adjusted.

## Competing interests

The author declares that they have no competing interests.
